# Self-employment and health inequality of migrant workers

**DOI:** 10.1186/s12913-022-08340-4

**Published:** 2022-07-21

**Authors:** Deshui Zhou, Xin Wen

**Affiliations:** 1 School of Finance and Public Management, Anhui University of Finance & Economics, 962 CaoShan Road, Bengbu City, 233030 Anhui Province China; 2 School of Public Administration, Dongbei University of Finance & Economics, 217 JianShan Street, Dalian City, 116001 Liaoning Province China

**Keywords:** Self-employment, Health inequality, Migrant workers, Public welfare

## Abstract

**Background:**

Self-employment is one of the most common forms of employment for migrant workers in China. However, migrant workers’ lifestyle and behavior, as well as health disparities among them, would be impacted by self-employment. This research aims to explore the mechanism and group differences of the effect of self-employment on health inequality among Chinese migrant workers.

**Materials and methods:**

To explore the effect of self-employment on health inequalities among migrant workers, this research uses the data from the 2018 China Migrant Workers Dynamic Monitoring Survey, and the RIF-I-OLS decomposition method.

**Results:**

We find that self-employment will reduce the health inequality of Chinese migrant workers significantly, especially among migrant workers with low education, low income, and low social integration. A further examination reveals that self-employment can directly promote the self-rated health of migrant workers. Additionally, it indirectly alleviates the health inequality among migrant workers by mediating effect of expanding access to public welfare, such as by establishing health records and strengthening health education.

**Conclusion:**

The government should permit and encourage migrant workers to engage in self-employment. It is necessary to provide public services such as health education, health records, and health rights for migrant workers, and focus on the employment of migrant workers in city, especially those with low income and low education. we believe that measures should be taken to enhance migrant workers’ sense of belonging in urban China Only on this basis can health inequality among migrant workers be truly reduced.

## Introduction

Health is an important human capital [1]. The average life expectancy worldwide is 73.3 years old, while the average healthy life expectancy worldwide is 63.7 years old, according to the World Health Statistics published by WHO in 2021. This indicates that although the life expectancy has been effectively increased, there is still room for improvement in terms of healthy longevity. Immigrants are a group with high mobility, making them more susceptible to the effects of uncertain social risks on their health [2, 3]. The health difference is ubiquity in modern society, and this difference can be attributed to both physical and socioeconomic features of an individual or group, with socioeconomic characteristics having a more pronounced effect. Social inequality is often associated with social stratification, O ‘Donnell et al. argue that health inequality should refer specifically to the health difference associated with economic income [4]. It is important to address how to preserve and improve the health of immigrants as well as the contributing factors of health inequality among immigrants.

As the largest developing country in the world, there are 285 million migrant workers in China. The migrant workers are a special group that emerged during China’s urbanization process and have made great contribution to urbanization and industrialization [5]. Although migrant workers typically live and work in cities, they often grow up in rural areas where they own land or homestead. The health of migrant workers, who make up a significant portion of the migrant population, is a key component of the Healthy China initiative, which may preserve social harmony and advance health equity [6]. At the same time, health is the foundation of migrant workers’ work and lives. It means that they are seeking greater health, happiness, security, and a sense of gain [7]. But the health of migrant workers, as a part of domestic migration, is a weak point in realizing *Healthy China* strategy [8]. The determinant of health inequality among migrant workers has not been fully explained by the academic circle, especially from the perspective of migrant workers’ self-employment. In our paper, we will make a fresh attempt to investigate how self-employment affects health inequality among migrant workers.

Why do we believe that this study will be a fresh effort? Self-employment is a key component of the entrepreneurship, and it includes the various migrant worker occupations found in non-farm sector, the self-employed workers can work for themselves as well as hire other people [9].Self-employment is a new way to foster employment, that can improve living condition and optimize the employment structure of migrant workers, and serve the crucial purpose of helping them to achieve a fuller and higher quality employment. Additionally, self-employment has an impact on the accumulation of migrant workers’ human capital and economic empowerment [10], as well as on health inequality among them. On the one hand, migrant workers will have more flexibility in their work schedules and a higher standard of living through self-employment, allowing people to build up their entrepreneurial skills and enhance their capacity to manage complex situations and risks [11], which is helpful in reducing health inequality. On the other hand, self-employment might not be the best option for migrant workers in the labor market.

Self-employed workers have to take business risks on their own due to limitations in their human and social capital, which adds some financial load and mental stress [12], and may have an expanding effect on health inequality among migrant workers. Therefore, it is difficult to draw a consistent inference on the relationship between self-employment and health inequality based on theoretical analysis. This offers a fresh perspective for our study, and we will use empirical analysis to evaluate the causal relationship between self-employment and health inequality among migrant workers.

Our inspiration comes from the observation of Chinese migrant workers. The researchers have studied the relationship between self-employment and health, which have laid the foundation for us, but there are still some controversies in these studies. For example, self-employment may help people achieve their work goals and sense of fulfillment [13, 14], as well as improve their health perception [15], and self-employed workers were tend to have much better health and work capacities than employees [16, 17]. However, Rietveld et al. believed that self-employment had a significant negative effect on health [18]. Although self-employment would increase job satisfaction but it could also result in some mental problems [12, 19, 20]. Meng and Xue believed that self-employment had a negative effect on the mental health of migrant workers [21]. The health instability of migrant workers in developing countries would be made worse by their involvement in unstable self-employment with low status and poor pay, such as street vendors, personal, and domestic services [22, 23].

There aren’t many studies on the relationship between self-employment and health inequality. Lewin found that the self-employed workers were more vulnerable to the threat of uncertainty and asset loss based on a study in Israel, and there was significant health inequality existed among them compared with the employed workers [22]. Based on the survey data of 32,630 workers aged 20–59 in South Korea, Kim et al. [9] found that the self-employed workers were more likely to have physical or mental health problems and much severer health inequality compared with the employed workers. And Kalleberg found that the proliferation of informal employment, like self-employment, had aggravated the inequality in Korea [24], the self-employed workers had a higher suicide rate [25] but poorer sleep quality [26] than employed workers in Korea. A Swedish study found that the self-employed workers had poor health, but there were lower health inequality among low-income self-employed workers than formal employment [27]. Krittanawong et al. concluded that self-employment was associated with high cardiovascular risk [28]. Schneck believed that self-employment was an important source of increasing income inequality in the labor market based on a German research [29]. Berkowitz and Novrinda showed that self-employed workers were more likely to be uncovered by health insurance [30, 31]. A Polish study concluded that there were different types of health difference between self-employed and employed workers, with self-employed workers being more susceptible to mental health issues while employed workers are more susceptible to physical health issues [32]. And Searson discussed the health difference between immigrants and non-immigrants [33].

In summary, self-employment, as an important form of employment for migrant workers, has a significant impact on their health. We find that there are many studies about determinants on health inequality among migrant workers after literature review but few studies about the impact of self-employment on this, and there is no further discussion on the internal mechanism or group difference. The key contributions in our study are outlined below, in contrast to earlier research: Firstly, we discuss the impact of self-employment on health inequality among Chinese migrant workers from the perspective of self-employment, which has some innovation on academic perspective. Secondly, we use RIF-I-OLS decomposition to assess health inequality, and we explore the mechanism of self-employment affecting health inequality by a mediating effect model, which is more in-depth in the research content. Thirdly, we identify the vulnerable groups of migrant workers with low income, low education and low social integration, and we examine the heterogeneity of self-employment on the health inequality in vulnerable migrant workers, which has extended the depth of research.

### Data sources and variable measures

#### Data sources

The data in this paper comes from 2018 *Dynamic Monitoring Survey Data of Migrant Workers* released by the Chinese National Health and Wellness Commission. The object of this survey is the floating population over 15 years old who have lived in the inflowing area more than a month without local household registration. The survey covers various aspects such as employment, mobility, household characteristics, income and expenditure, as well as health of the floating population. And the data covers 31 provinces, autonomous regions and municipality directly under the Central Government, with a sample size of 150,000, which is broadly representative of the whole country, thus it can examine the occupational characteristics and health status of the migrant population in all aspects. This paper mainly focuses on the migrant workers in the floating population, so we choose the floating population with agricultural household registration in this data (including Farmers to Citizens) as the research object, and limits the age of migrant workers between 17 and 59 years old, after screening all variables, the sample size is 86,438.

#### Measurement on health inequality of migrant workers

In this paper, the dependent variable is the health inequality among migrant workers. But health inequality is not a variable which can be directly measured, it needs to be measured by inequality index using individual health indicators. There are different measurements of individual health, and we choose the Self-Rated Health in this paper, which is shown as “How do you feel about your health?“in the questionnaire, and we put the answer “Healthy” is assigned to 4,“Basically healthy” = 3, “Unhealthy, but I can take care of myself” = 2, “Unable to take care of myself”= 1;and this health variable is a positive variable. Health inequality is usually measured using the Gini coefficient and the concentration index, we use the concentration index to measure the degree of health inequality. The concentration index requires that the health variable is a continuous variable between 0 and 1, so the Self-rated Health variable needed to be standardized. And the health inequality in our study is the inequality which is related to economic income, so we need a rank variable to measure the deviation of individuals’ economic income in sample, and the economic income variable is selected as “How much did you earn last month (or last employment)?”, and the rank variable *R* is the fractional rank of individuals in the sample sorted by per capita income from low to high. The specific method is as follows:

First, the treatment of the dependent variable. We use Donnell’s methods to indirectly standardize the self-rated health variable by an ordered probit model [4], this will adjust the self-rated health variable to a continuous variable in [0–1] interval. Before measuring the health inequality, it is necessary to construct a production function equation of individual health. Our individual health variable is the self-rated health Sah*, which is an ordered categorical variable, values from 1 to 4, and the basic regression model is set as the ordered probit model:


1


Sah_i_*in eq. (1) is the self-rated health of individual *i*, δ is the cut point to be estimated, and Sah_i_ is the potential continuous variable of Sah_i_*, which can be expressed by the equations with a series of explanatory variables.

2 $${Sah}_i=\alpha +\beta {x}_i+{\varepsilon}_i$$

In eq. (2), Sah_i_ is the latent variable of self-rated health of individual *i*, x_i_ is all explanatory variables, Ɛ_i_is the error term, α is the constant term and β is the regression coefficient.

According to the health equation of self-rated health Sah_i_ to explanatory variables x_i_, we can estimate the predicted value of Sah_i_ based on eq. (2), named ${\hat{\mathrm{Sah}}}_{\mathrm{i}}$, and the range of ${\hat{\mathrm{Sah}}}_{\mathrm{i}}$ is (−∞,∞), and then we make it normalization by dispersion standardization to get H_i_:

3 $${H}_i=\frac{{\hat{Sah}}_i-\mathit{\min}{\hat{Sah}}_i}{\mathit{\max}{\hat{Sah}}_i-\mathit{\min}{\hat{Sah}}_i}$$

Second, the treatment of the rank variable. The rank variable (R) is the fractional rank of the individuals sorted by economic income in the sample, which measures the degree of deviation of the economic income ranking in the sample, and R is the order of the i-th person in the sample sorted by his or her income. The formula is:

4 $${R}_i=\frac{i-0.5}{n}$$

where *i* represents the i-th individual in the sample, and *n* is the sample size.

#### Explanatory variables and control variables

The core explanatory variable in this paper is self-employment, which is defined as employer or self-employed economy. The corresponding question is “What is your current employment status?”, we assign a value of 1 to migrant workers who answered “self-employed” and “I am an employer”, and 0 to those who answered “be employed”. According to Grossman’s health demand theory, health is a crucial component of human capital that not only improves labor productivity effectively but also offers healthy “labor time” for continuous production activities [34]. Grossman studied health as a function of age, gender, education, income, medical services or individual behavior, and health is affected by personal characteristics as well as family characteristics and other socioeconomic characteristics. Based on this theory, combined with the characteristics of the migrant workers, we have considered the research of Rietveld et al. [18] as a reference and set our control variable using as many of the individual characteristics, family characteristics, work characteristics, and regional characteristics of migrant workers as possible. The demographic variables are the well-known aspects that affect everyone’s health status, such as gender, age, and education (Bener A., 2017) [35]. These variables are the main focus of the control variables of personal characteristics. The control variables of family characteristics are mainly about the family life of migrant workers, employment and health of individual migrant workers is related to their family life and family pressure, which includes marriage, number of children, family size and monthly household income (Zhang S. et al.,2020) [36]. The control variables of work characteristics are about the difference of migrant workers in working environment, including whether migrant workers moved across provinces, length of mobility, work hours in a week and medical insurance. The control variables of regional characteristics mainly focuses on the differences of the regional development level between regions in China, including living in the eastern, midland, western and northeastern regions. The control variables are shown in Table 1.

**Table 1:** Descriptive statistics of variables

Variable	Variable Definitions	Mean	Standard Error
Dependent Variable			
Self-rated Health	“Healthy” =4, “Basically healthy” = 3, “Unhealthy, but I can take care of myself” = 2, “Unable to take care of myself “= 1.	3.881	0.351
Explanatory Variables			
Self-employment	Self-employment =1,others = 0	0.444	0.497
Personal Characteristics			
Gender	Man = 1,Women = 0	0.566	0.496
Age	Continuous variable of Age	37.360	8.712
Education	primary school and below = 1,junior high school =2,high school =3,university and above =4	2.250	0.887
Political status	Communist =1,others = 0	0.037	0.188
Family Characteristics			
Marriage	First marriage & remarriage =1, Divorce, widowhood, cohabitation =0	0.961	0.194
Number of Children	Number of children in the respondent’s family	1.536	0.750
Family Size	Total number of respondents’ households	3.471	0.958
per capita income	The average monthly income level of the respondents ‘families	7.609	0.601
Work Characteristics			
Inter-provincial mobility	Inter-provincial mobility =1,others = 0	0.518	0.500
length of mobility	Time to live in a mobile area (years, continuous variable)	7.618	6.085
Work Hours	Working hours per week, continuous variable	60.710	20.123
Medical Insurance	With a medical insurance =1,without = 0	0.952	0.213
Regional Characteristics			
Eastern	Living in the eastern = 1,Others = 0	0.469	0.499
Western	Living in the western = 1,Others = 0	0.280	0.449
Northeastern	Living in the northeastern =1,Others = 0	0.053	0.223
Mediating Variable			
Health Record	Without health record, and never heard of it = 1,without established a health record, but have heard of it =2,Established a health record =3	2.633	1.022
Health Education	Have received health education =1,haven’t = 0	0.815	0.389

Descriptive statistical result shows that, the percentage of self-employed migrant workers who consider themselves to be “healthy” is higher than the percentage of employed migrant workers, although there is no statistically significant difference in the distribution of self-rated health between the two groups. According to the further descriptive statistics about the degree of health inequality between the two groups, the health concentration index of the self-employed migrant workers group is 0.0072, the employed migrant workers group is 0.0078. This implies that there is a pro-rich health inequality among migrant workers, but the health inequality is less pronounced among self-employed migrant workers than it is among employed migrant workers.

## Measurement methods

### Calculation of concentration index

This paper uses concentration index to measure the health inequality among migrant workers. Based on the above calculation of the health and income rank variable, according to Donnell’s method [31], the health concentration index (CI) is expressed as:

5 $$CI=\frac{1}{n}{\sum}_{i=1}^n\frac{H_i\left(2{R}_i-1\right)}{\overline{H}}$$

where H_i_ is the health of individual *i*, R_i_ is the rank of income, $\overline{\mathrm{H}}$ is the mean of the health variable H_i_, *n* is the sample size. The value of health concentration index CI is in the range of [− 1,1], and the health variable in this paper is a positive variable, that is, the larger the value, the better the health of the individual. So, when CI > 0, the group with higher income has better health, means health inequality is expressed as “pro-rich”. When CI < 0, health inequality is expressed as “pro-poor”. When CI = 0, means that there is no health inequality.

### RIF-I-OLS decomposition

According to eq. (5) above, we have calculated the degree of health inequality by the concentration index. Then we need to decompose the concentration index to examine the effect of various factors on health inequality. We use the RIF-I-OLS decomposition method, this method is a regression decomposition based on the recentered influence function, the idea of this decomposition method is to use RIF’s estimation of the concentration index, and then establish a connection between the concentration index and the explanatory variable, and thus achieve regression decomposition. It can be achieved in two steps:

Firstly, calculate the RIF value of the concentration index.

6 $$RIF\left(h,R;{\nu}^{CI}\right)={\nu}^{CI}\left({F}_{h,R}\right)+ IF\left(h,R;{\nu}^{CI}\right)$$

where ν^CI^ represents the function of the concentration index. Then, the RIF value can be regarded as dependent variable, and we can put it in the regression.

$$E\left[ RIF\left(h,R;{\nu}^{CI}\right)\right]=E\left[{\nu}^{CI}\left({F}_{h,R}\right)+ IF\left(h,R;{\nu}^{CI}\right)\right]={\nu}^{CI}\left({F}_{h,R}\right)$$

7 $${\nu}^{CI}\left({F}_{h,R}\right)=E\left[ RIF\left(h,R;{\nu}^{CI}\right)\right]={E}_x\left\{E\left[ RIF\left(h,R;{\nu}^{CI}\right)|X=x\right]\right\}={E}_x\left({\beta}^TX+\varepsilon \right)={\beta}^TX$$

Based on rational choice theory, individual social activity will typically follow the presumption of cost minimization and revenue maximization [37]. Likewise, the working population makes decisions based on rational choice theory in their career choices, migrant workers also make dicision about their career choice based on the potential revenue. Because of the precarious status in the labor market, the flexibility of employment and the diversity of welfare sources will be encouraged when migrant workers can overcome the “lock” state in employment and choose self-employment. Currently, self-employment pays more than traditional work in terms of revenue. Moreover, the benefits of self-employment are not only reflected in their income, existing literature points out that self-employment can also promote health and improve public welfare [38]. When migrant workers applying for employment in the labor market, and their choice of self-employment is a rational choice based on the principle of revenue maximization. Migrant workers believe that they can have more income and higher accessibility of public welfare through self-employment, and then reducing the health inequality.

## Empirical results

### The health inequality on self-employment of migrant workers and decomposition

To examine the impact of self-employment on health inequality among migrant workers, we use the RIF-I-OLS decomposition method to estimate the RIF value of the concentration index and analyze the health inequality results from subtle changes from the health distribution, which is able to examine the impact of changes in explanatory variables on health inequality. Table 2 presents the decomposition on health inequality by RIF-I-OLS method, model *a* to model *d* shows the decomposition results by adding control variables in turn. The result shows that self-employment has a significantly negative effect on the health inequality among migrant workers, indicating that when migrant workers are more likely to choose self-employment, the degree of health inequality will decrease. However, the result in Table 2 shows that the coefficient of self-employment is still small, indicating that although self-employment has a statistically significant effect on health inequality among migrant workers, the change of self-employment can hardly make a large impact on an objective fact that health inequality among migrant workers do exist objectively.

**Table 2:** Impact of self-employment on health inequality among migrant workers: RIF-I-OLS decomposition method

Variable	a	b	c	d
Self-Employment	−0.0017***	−0.0015***	− 0.0009	−0.0013**
	(0.0005)	(0.0005)	(0.0006)	(0.0006)
Gender	−0.0017***	− 0.0017***	− 0.0015***	− 0.0016***
	(0.0005)	(0.0005)	(0.0005)	(0.0005)
Age	−0.1388***	−0.1392***	− 0.1493***	−0.1480***
	(0.0110)	(0.0112)	(0.0112)	(0.0112)
Age square,	0.1630***	0.1602***	0.1669***	0.1653***
	(0.0105)	(0.0107)	(0.0108)	(0.0108)
Education	−0.0067***	− 0.0030***	− 0.0044***	− 0.0042***
	(0.0010)	(0.0010)	(0.0010)	(0.0010)
Political status	0.0004	0.0007	0.0006	0.0005
	(0.0014)	(0.0014)	(0.0014)	(0.0014)
Marriage		−0.0053***	− 0.0054***	− 0.0053***
		(0.0014)	(0.0014)	(0.0014)
Number of children		0.0133***	0.0147***	0.0165***
		(0.0035)	(0.0035)	(0.0035)
Family size		−0.0172***	−0.0172***	− 0.0160***
		(0.0035)	(0.0035)	(0.0035)
Per Capita Income		− 0.0708***	−0.0662***	− 0.0583***
		(0.0057)	(0.0057)	(0.0059)
Inter-Provincial Mobility			−0.0025***	− 0.0019***
			(0.0005)	(0.0006)
Length of Mobility			0.0186***	0.0184***
			(0.0026)	(0.0026)
Work Hours			−0.0096***	−0.0092***
			(0.0017)	(0.0017)
Medical Insurance			−0.0048***	−0.0048***
			(0.0012)	(0.0012)
Eastern				−0.0011
				(0.0008)
Western				0.0026***
				(0.0008)
Northeastern				0.0035***
				(0.0013)
Constant	0.0195***	0.0718***	0.0800***	0.0732***
	(0.0017)	(0.0044)	(0.0046)	(0.0047)
R-squared	0.0109	0.0131	0.0145	0.0150
Sample Size	86,438			

Using RIF-I-OLS model to estimate

***, **, * are statistically significant at 1,5,10%, robust standard error in parentheses

We also pay attention to the other variables. In individual characteristics, male migrant workers have low health inequality compared to female, indicating that there is a gender health difference and female migrant workers are more vulnerable to health risks. The health inequality among migrant workers increases significantly with increasing age, and in order to observe whether there are nonlinear relationships between health inequality and age, the age square has been brought into baseline regression in this study. The result shows that age has significantly negative effect on health inequality while age square has significantly positive effect on it, indicating that a U-shaped relationship is found between health inequality and age in immigrant workers, that is the degree of health inequality tends to decline with the increase of individual age, but the effect of age on reducing health inequality will be weakened with the further increase of age. High education of migrant workers has low health inequality, but communist variable has no significant effect on the health inequality among migrant workers. In family characteristics, the degree of health inequality is relatively lower among married migrant workers, the degree of health inequality among migrant workers is higher when a family has more children, and a larger family size and higher household income will reduce the degree of health inequality. In job characteristics, inter-provincial mobility significantly reduces the degree of health inequality, but as migration times lengthen, migrant workers will face higher health inequality. The health inequality is worse when migrant workers have longer work time, and medical insurance has significantly decreased the health inequality among migrant workers. In regional characteristics, the degree of health inequality is low when migrant workers living in eastern, but the degree of health inequality is high when migrant workers living in western and northeastern region.

### Robustness test

We conduct robustness test from the following three aspects:

1. Eliminating the impact of urban differences. The floating population usually migrates to large cities. Since Chinese cities can be classified according to their size and level of economic growth, we exclude the sample from megacities like Beijing, Shanghai, Guangzhou, and Shenzhen. Model (1) in Table 3 shows that migrant workers’ choice of self-employment can still significantly reduce the degree of health inequality after excluding four megacities.
2. Excluding outliers.5% truncated tails before and after the income variable. Outliers may produce false results, the regression curve may deviate from the true trend, and then the relationship between the variables may be incorrectly affected. Since health inequality is income-related inequality, this paper makes a truncation of the income rank of migrant workers by 5% before and after, the result of model (2) in Table 3 shows that the effect of self-employment on health inequality is still significantly negative.

**Table 3:** Robustness test

Variable	(1)	(2)
Self-Employment	−0.0017***	−0.0026***
	(0.0006)	(0.0006)
Opportunity Self-Employment		
Subsistence Self-Employment		
Other Variables		
Constant	0.0578***	0.0334***
	0.0049	0.0051
R-squared	0.0132	0.0070
Sample Size	77,463	80,801

Using RIF-I-OLS model to estimate

***, **, * are statistically significant at 1,5,10%, robust standard error in parentheses

3. Instrumental variable method.

Because of unobservable factors and reverse causality, and which may lead to endogenous problems. However, the RIF decomposition based on Recentered influence function cannot directly examine the effect of self-employment on health inequality in migrant workers by instrumental variable method, so we attempt to overcome endogenous problems by instrumental variable method to examine the effect of self-employment on health status in migrant workers. We choose an interaction as an instrumental variable of IV estimation for endogenic correction, this interaction is between self-employment rate of migrant workers in each county and changing range of age, and the changing range of age refers to the ratio between the age of first out-migration and the current age of each migrant worker. Table 4 displays the results of the instrumental variable model, after controlling all of the control variables, the instrumental variable is significantly positive at the 1% level in the IV regression. It implies that the instrumental variables have a significant positive effect on the explanatory variables of this paper, and the robustness of the regression results has been confirmed.

**Table 4:** Endogeneity test based on CMP Model

	IV	Self-Employment
First Stage Regression:	1.0319*** (0.0092)	
Second Stage Regression:		0.2093*** (0.0356)
Other Variable	control	
Weak Iv Test	AR:29.00***	
	Wald:29.03***	
Sample Size	86,438	

Using CMP model to estimate

***, **, * are statistically significant at 1,5,10%, robust standard error in parentheses

### Heterogeneity

Individuals with different characteristics will have different levels of health, so this paper compares the educational, financial, and social integration levels of migrant workers in a sub-sample. According to the education of migrant workers, migrant workers are categorized into three levels: (1) elementary school and below, (2) junior high school, (3) senior high school and above. According to the income of migrant workers, reflected in the questionnaire as “How much was your personal income last month?“The data shows that migrant workers’ sample average monthly salary was RMB 4796.85. Based on this, this paper divides the samples of migrant workers into two groups: (1) high income migrant workers, and (2) low income migrant workers. Samples with incomes above RMB4796.85 are classified as high income migrant workers. According to the social integration of migrant workers, this paper uses the residence willingness of migrant workers to live in inflow area as proxy variable, which is reflected in the questionnaire as “If you intend to stay in this city, how long do you expect to stay?“We put the answer “I don’t want to stay here” and “I have no idea” are assigned to 0, “I plan to stay here, but I don’t know how long”=1, “I will stay here for 0-4years”=2, “I will stay here for 5-9years”=3, “I will stay here for more than 10years”=4, “I plan to settle here”=5. According to this, we have generated a social integration variable, (1) high social integration is defined as residence willingness for more than 5 years, and (2) low social integration is defined as residence willingness for less than 5 years.

Model *a* in Table 5 is a sub-sample test by education. The RIF-I-OLS decomposition result shows that self-employment can significantly reduce the health inequality among migrant workers with primary education and below, but it is not significant for the other three subsamples. Model *b* in Table 5 is a sub-sample test by income. The result shows that self-employment can significantly reduce the health inequality among migrant workers with low-income, but this effect in high-income migrant workers is positive but not significant, indicating that the increase of the probability of migrant workers choosing self-employment can significantly reduce the degree of health inequality among low-income migrant workers. Model *c* in Table 5 is a sub-sample test by social inclusion. The RIF-I-OLS decomposition result shows that self-employment has a significant negative effect on health inequality among migrant workers with low social inclusion, but has no significant effect on migrant workers with high social integration. The above results show that self-employment can alleviate the health inequality among migrant workers, and particularly significant in migrant workers with low-education, low-income and low social inclusion.

**Table 5:** Heterogeneity

Variable	a. by education			b. by income		c. by social inclusion	
	(1)	(2)	(3)	(1)	(2)	(1)	(2)
Self-Employment	−0.0043**	0.0002	−0.0014	0.0006	−0.0017**	− 0.0012	− 0.0024***
	(0.0017)	(0.0007)	(0.0008)	(0.0007)	(0.0008)	(0.0008)	(0.0008)
Other Variable	Control	Control	Control	Control	Control	Control	Control
Constant	0.0427***	0.0540***	0.0216***	0.0029	0.0645***	−0.0012	0.0492***
	(0.0126)	(0.0063)	(0.0068)	(0.0064)	(0.0067)	(0.0008)	(0.0064)
R-squared	0.0086	0.0064	0.0078	0.0006	0.0105	0.0162	0.0100
Sample Size	15,983	42,754	27,701	33,963	52,475	41,907	44,531

Using RIF-I-OLS model to estimate

***, **, * are statistically significant at 1,5,10%, robust standard error in parentheses

## Further discussion

### The effect of self-employment on self-rated health of migrant workers

The effect of self-employment on migrant workers’ self-rated health is covered in this section. Table 6 shows the effect of self-employment on health of migrant workers using the ordered probit model. Models (1) to (4) show the regression results of adding control variables in turn. The results show that after adding the control variables gradually, self-employment has a significant positive effect on health of migrant workers, indicating that when migrant workers are inclined to choose self-employment, they will have a health improvement either. The conclusion is that self-employment can significantly promote the health of migrant workers.

**Table 6:** The effect of self-employment on migrant workers’ health

Variable	(1)	(2)	(3)	(4)
Self-employment	0.0509*** (0.0115)	0.0468*** (0.0116)	0.0849*** (0.0125)	0.0988*** (0.0128)
Individual Characteristics	control	control	control	control
Family Characteristics	–	control	control	control
Job Characteristics	–	–	control	control
Regional Characteristics	–	–	–	control
Pseudo R2	0.0425	0.0466	0.0497	0.0510
Sample Size	86,438			

Using ordered probit model to estimate

***, **, * are statistically significant at 1,5,10%, robust standard error in parentheses

Chinese migrant workers, in contrast to those from other developing countries, are more likely to accumulate wealth and acquire capital by engaging in wage employment before transitioning to self-enterprise. Migrant workers usually engage in heavy physical hired work in wage employment, while self-employment may lead to high income and free working environment [39]. According to Grossman’s health demand theory [1],self-employment by migrant workers can lessen the health depletion from work and have a positive impact on health. This is in contrast to traditional employment with its poor environment and high labor intensity.

### Health inequality of migrant workers with different types of self-employment

In this paper, the explanatory variables are divided into subsistence self-employment and opportunity self-employment base on employment status. Opportunity self-employment is defined as hiring at least one worker, corresponding to “employers”, subsistence self-employment is defined as without hiring others, corresponding to “self-employed workers”. The result in Table 7 shows that both opportunity self-employment and subsistence self-employment have a significant negative effect on health inequality, with opportunity self-employment having a greater impact than subsistence self-employed migrant workers. The difference between opportunity self-employment and subsistence self-employment could be explained by the fact that opportunity self-employment is at a higher stage of development. Compared with subsistence self-employment, the opportunity self-employment have advantages on development direction, the income and social security obtained in the work, so they will have a stronger ability to invest in their own health and have more advantages in obtaining health resources.

**Table 7:** Health inequality among migrant workers with different types of self-employment

Variable	(1)	(2)
Opportunity Self-Employment	−0.00196**	
	(0.000963)	
Subsistence Self-Employment		−0.000997*
		(0.000583)
Other Variable	control	control
Constant	0.0539***	0.0554***
	(0.00459)	(0.00453)
R-squared	0.0122	0.0122
Sample Size	86,438	86,438

Using RIF-I-OLS model to estimate

***, **, * are statistically significant at 1,5,10%, robust standard error in parentheses

### Mechanism analysis of self-employment affecting health inequality of migrant workers

The empirical result above shows that the increase of the probability on migrant workers choosing self-employment can reduce the degree of health inequality, this section attempts to discuss the mechanism of this effect. We choose the accessibility of public welfare obtained by migrant workers as a mediator variable, and divides it in two pathways: acceptance of health education and the establishment of health records. As a group uses physical labor for a living, migrant workers are vulnerable to diseases and usually not well-educated, it is necessary to provide them with health education by communities and work, such as the prevention of occupational disease, infectious disease and chronic disease, the reproductive health, maternal and child health, mental health, self-help of public emergencies, and other aspects of health education [40]. At the same time, the establishment of health records is a new production with Internet technology development in recent years. Health records provide information about residents’ health in addition to being a tool for documenting illnesses and medical procedures. In order to carry out various health care tasks and satisfy the needs of the floating population for health services by community, such as prevention, medical and health, rehabilitation, health education, fertility guidance, it is crucial to establish health records. It is also a fundamental step in improving the accessibility of basic public services in health and family planning for the floating population.

Table 8 shows the results of mediating effect test using the health education variable as a mediator variable, the results reflect that the increasing probability of migrant workers choosing self-employment can improve the probability of receiving health education, and health education of migrant workers can make them acquire more health knowledge so that they can have better protection for themselves at work, which is conducive to promote individual health [41, 42] and reducing the occurrence of health inequality. At the same time, self-employment also encourages migrant workers to use and understand their health records, and the establishment of health records can observe the changes of individual health and analyze the trend of disease development and treatment effect, which is conducive to health care decision-making and maintenance of individual health. Health records, as a basic support measure of the demand for prevention and medical care, can meet the basic needs of residents while also providing more significant health promotion for migrant workers with lower incomes, thereby reducing health inequalities.

**Table 8:** Mechanism analysis of self-employment affecting health inequality

Variable	Mediator Variable		Dependent Variable: Health Inequality	
	Health Education	Health Record		
Self-Employment	0.0865***	0.04073***	−0.00155***	−0.00167***
	(0.0112)	(0.00331)	(0.000571)	(0.00060)
Health Education			−0.00365***	
			(0.000674)	
Health Record				−0.00253***
				(0.00064)
Other Variables	control	control	control	control
Constant	1.217***	0.61086***	0.0575***	0.06033***
	(0.0928)	(0.02733)	(0.00458)	(0.00488)
R-squared	0.0333	0.0377	0.0126	0.0133
Sample Size	86,438			

Using RIF-I-OLS model to estimate

***, **, * are statistically significant at 1,5,10%, robust standard error in parentheses

Migrant workers have contributed significantly to China’s industrialization and urbanization. But most migrant workers in cities are engaged in high-intensity work, which damages their health seriously. With the gradual relaxation of some restriction policies on the floating population in cities, the health status of migrant workers has been prioritized and improved over the past several years. But at the same time, the health difference in migrant workers has emerged, different job status and employment relationships are reasons for health inequality among migrant workers. So it is necessary to take measures to protect the health of migrant workers and promote health equality among them. Additionally, it is a significant step in improving the accessibility of migrant workers’ public health welfare and promoting a more equitable health system for them. Health and health inequality are influenced by a variety of factors, including genetic and inherited factors, the physical environment, individual behavior and habit, as well as the provision and demand for public health services [43, 44]. Consequently, risk management and equitization of public welfare can be used to intervene in health inequality. By providing health education and establishing health records for the migrant workers, it can not only provide health knowledge, correct their unhealthy lifestyles and reduce the incidence of diseases**,** it can also intervene, assess and manage their health and diseases, identify potential health risks and respond to health needs in a scientific and technological manner, which will improve the health status of migrant workers and alleviate the health inequality among them.

## Conclusion and suggestion

Modern industrialized societies place a premium on health and health disparities among migrant workers, and addressing these issues is essential to implementing *Healthy Chinese* Strategy. This paper uses the 2018 China Migrant Workers’ Dynamic Monitoring Survey Data and examines the impact of self-employment on health inequality among migrant workers through the concentration index and RIF-I-OLS decomposition methods. We find that social inequality is a problem that is frequently present in today’s social environment, and that migrant workers experience pro-rich health disparity in addition to the upper class’ constant dominance of social resources. As a new way of employment that is more competitive and entrepreneurial, while self-employment can significantly improve the health of migrant workers, and narrow the gap of resource distribution, we find that it is also an important reason in reducing the degree of health inequality among migrant workers. There is a statistically significant decline in the degree of health inequality that is related to migrant workers’ rising preference for self-employment. Further analysis shows that the welfare acquisition plays an important role in migrant workers’ health. Specifically speaking, self-employment will alleviate the health inequality among migrant workers by mediating the effect of improving the accessibility in public services, such as promoting health education and establishing health records. In addition, there are vulnerable groups among migrant workers, and the mitigation effect of self-employment on migrant workers’ health inequality is more significant among migrant workers with low-education, low-income, and low social integration.

The following recommendations are based on the aforementioned findings. First and foremost, migrant workers should be allowed and supported to engage in self-employment activities, they should also receive policy protection and financial support, as they will enable migrant workers improve their capacity for personal growth. Self-employment entry limitations with a high threshold should not be implemented, such as providing policy support for migrant workers to carry out self-employment activities in inflow areas, providing business suitable worksite and environment, and providing special financial support. Second, the government needs to move more quickly toward achieving public service parity between urban and rural areas, provide migrant workers with public services like health education, health rights, and health records, and increase their accessibility to social welfare by encouraging the expansion of urban public services. For instance, expanding the construction of health records, keeping track of changes in health status, dynamically monitoring the history of disease occurrence and potential health risks, and providing migrant workers with the same health rights and interests as urban residents. Furthermore, the entire society needs to pay more attention to the employment of low-income and low-education migrant workers, provide multiple employment options to improve their self-employment selectivity, and provide them with skills training for employment. For example, migrant workers should be provided various entrepreneurial and risk knowledge training in order to improve the quality of their employment, their chances of success, and their capacity for risk avoidance. And knowledge will bring more opportunities for self-employment activities and improve the success rate, and then their investment in healthy human capital will be improved. Finally, it is essential to address the systemic issue of migrant workers’ social mobility, particularly by removing the barriers to their children’s education and household registration. This is the critical way to enhance migrant workers’ sense of belonging in the city and realize the great goal of *Urbanization of Human Beings*.

It is difficult to get continuous tracking data due to the high mobility of migrant workers, so this study uses the cross-sectional data of CMDS in 2018 and it is necessary to mine the tracking data of migrant workers in the follow-up study. At the same time, the influence of migrant workers choosing self-employment on health inequality is also estimated in this article; however due to the differences in health inequality across migrant workers, it is also worthwhile to discuss the health inequality within self-employed groups. This topic wasn’t explored in full in our paper, which is also a direction for further research.

## Data Availability

The data that support the findings of this study are available on request from the corresponding author upon reasonable request. The data are not publicly available due to privacy or ethical restrictions. If there is any need, please contact the corresponding author at any time (corresponding author*Xin Wen: wenxin950520@163.com).
